# Comparing outcomes of TAVI and SAVR in low or intermediate risk symptomatic severe aortic stenosis: a systematic review and meta-analysis

**DOI:** 10.1515/med-2026-1466

**Published:** 2026-07-07

**Authors:** Khalid Rashid, Ahmedyar Hasan, Mustafa Javaid, Farrukh Ansar, Yahya Khan, Muhammad Hazqeel Kazmi, Taimur Farid, Faraz Waheed, Leena Ali, Salah Ali Saleh, Raza Ali Akbar, Muhammad Aamir Waheed

**Affiliations:** North Cumbria Integrated Care NHS Foundation Trust, Carlisle, UK; University of Minnesota, Minneapolis, MN, USA; Oxford University Hospitals, Oxford, UK; Alkhidmat Raazi Hospital, Rawalpindi, Pakistan; Institute of Kidney Disease, Peshawar, Pakistan; Sunderland Royal Hospital, Sunderland, UK; Ninewell Hospital, Dundee, Scotland, UK; Hayatabad Medical Complex, Peshawar, Pakistan; Nowshera Medical College, Peshawar, Pakistan; Hamad General Hospital, Doha, Qatar; Hamad General Hospital, Doha, Qatar; Hamad General Hospital, Qatar University, Doha, Qatar

**Keywords:** aortic stenosis, transcatheter aortic valve implantation, surgical aortic valve replacement

## Abstract

**Objectives:**

To compare the mortality and clinical outcomes of transcatheter aortic valve implantation (TAVI) versus surgical aortic valve replacement (SAVR) in patients with low- or intermediate-risk symptomatic severe aortic stenosis through an updated systematic review and meta-analysis of randomized controlled trials.

**Methods:**

This systematic review was performed using PubMed, Medline, Embase, and Cochrane databases for RCTS between 2017 and 2024 which evaluated mortality outcomes in patients with low to intermediate risk AS who received TAVI vs. SAVR. Relative risks (RRs) with 95 % confidence intervals (CIs) were pooled using random effects models.

**Results:**

TAVI showed a numerically lower but statistically insignificant mortality risk compared to SAVR (RR: 0.88, 95 % CI: 0.62–1.25, p=0.49). TAVI was associated with a higher risk of PPM insertion (RR: 2.19, 95 % CI: 1.55–3.16, p<0.001, i2: 80 %), aortic valve re-intervention (RR: 2.51, 95 % CI: 1.40–4.51, p<0.001; I^2^=0 %), new-onset bundle branch block (RR: 2.10, 95 % CI: 1.21–3.67, p<0.01; I^2^=84 %), and vascular site complications (RR: 4.77, 95 % CI: 2.11–10.81, p<0.001; I^2^=62 %). Atrial fibrillation incidence was lower with TAVI (RR: 0.29, 95 % CI: 0.23–0.38, p<0.001; I^2^=73). There was no significant difference in the rates of post-procedural myocardial infarction, stroke and endocarditis between TAVI and SAVR.

**Conclusions:**

TAVI has mortality outcomes comparable to SAVR in low to intermediate risk patients. TAVI offers advantages in atrial fibrillation reduction, but is associated with increased risk of PPM insertion, vascular complications, new-onset bundle branch block, aortic valve re-intervention. Individualized treatment decisions remain important for optimizing outcomes in low to intermediate risk patients.

**Prospero registration number:**

CRD42024547882.

## Key message

What is already known on this topic:

TAVI and SAVR have comparable mortality outcomes in low or intermediate risk aortic stenosis patients, but evidence on secondary outcomes like pacemaker implantation and vascular complications remain limited.

What this study adds:

This study incorporates the most recent randomized controlled trials to provide a clearer understanding of trade-offs between TAVI and SAVR in low to intermediate risk patients. It also highlights TAVI’s higher risks of pacemaker insertion, vascular complications, and re-intervention, alongside a lower risk of atrial fibrillation.

How this study may affect research, practice, or policy:

The findings emphasize the need for individualized treatment decisions, balancing TAVI’s benefits and risks. Future research should focus on long-term outcomes to improve our understanding of TAVI’s safety and efficacy.

## Introduction

Aortic stenosis (AS) is a progressive and potentially life-threatening condition, characterized by the narrowing of the aortic valve. This valvular heart disease is predominantly seen in the elderly, as it affects approximately 5 % of individuals over 65 years old [1]. The importance of effectively managing AS cannot be overstated, as symptomatic patients face high risks of morbidity and mortality, with a significant decline in survival rates if left untreated [1]. Presentations are variable, and the American College of Cardiology (ACC) provides a staging system to classify severity according to aortic valve area (AVA), transaortic velocity, and mean aortic pressure gradient [2]. Traditionally, the mainstay treatment of symptomatic AS has been surgical aortic valve replacement (SAVR) [3]. However, treatment strategies are constantly being reevaluated with the advent of less invasive options, such as transcatheter aortic valve Implantation (TAVI).

In 2012, the FDA approved TAVI in AS patients with high surgical risk [4]. TAVI has since emerged as the gold standard treatment for this subset of patients. Moreover, TAVI has become an accepted treatment modality for a broad spectrum of patients, including those with intermediate and low-risk profiles [5]. Despite its benefits, TAVI is not without its complications. Concerns persist regarding issues such as paravalvular leakage (PVL), conduction disorders (CD), and the risk of cerebrovascular events, all of which can significantly impact both short-term and long-term patient outcomes [6]. These potential complications underscore the necessity for a comprehensive evaluation of TAVI’s safety and efficacy in a broader patient population, particularly those at low and intermediate risk.

Given the recent publications of multiple randomized controlled trials (RCTs), which analyze the survival benefits of TAVI compared to SAVR in patients with symptomatic AS (at low and intermediate risk) [7], [8], [9], [10], [11], [12], [13], there is a pressing need to synthesize this data. This systematic review and meta-analysis aims to review and analyze the current literature to compare the mortality benefits of TAVI and SAVR in this patient population. We seek to provide a clearer understanding of whether TAVI should be considered the gold standard treatment for low and intermediate-risk patients with symptomatic AS, thereby guiding future clinical decision-making and optimizing patient outcomes.

## Methodology

The current study was registered at the International Prospective Register of Systematic Reviews (PROSPERO) after being reported in compliance with the published Preferred Reporting Items for Systematic Reviews and Meta-Analyses (PRISMA) guidelines. The full protocol is accessible at: https://www.crd.york.ac.uk/PROSPERO/view/CRD42024547882. No deviations from the registered protocol occurred.

## Search strategy

A systematic search of PubMed, MEDLINE, Embase, and the Cochrane Library was performed on May 26, 2024. Searches were restricted to randomized controlled trials, human studies, and English-language publications. The search strategy was devised following the PICOS scheme (explained later) to retrieve pertinent data from digital databases. In the final sample, 7 studies (from a total 614 records) met the eligibility criteria. A search query was formulated for each of the databases, using appropriate MESH terms, and terminologies. Two independent reviewers assessed all the studies for inclusion, and disputes were resolved with consensus. A summary of the databases and their respective search strategies are mentioned in the table below (Table 1).

**Table 1:** Description of search strings used for various databases in the literature search.

MEDLINE	((“Aortic Stenosis” [Mesh] OR “Aortic Valve Stenosis” [Mesh]) AND (“Surgical Aortic Valve Replacement” [Mesh] OR SAVR [Text Word)) OR (“Transcatheter Aortic Valve Replacement” [Mesh] OR TAVI [Text Word] OR TAVR|Text Word)
Cochrane library	(MeSH terms: (Aortic Stenosis” OR “Aortic Valve Stenosis”)) AND (MeSH terms: (“Surgical Aortic Valve Replacement” OR “Transcatheter Aortic Valve Replacement”)) OR (SAVR OR TAVI OR TAVR)
EMBASE	(Exp aortic stenosis/or “Aortic Stenosis” [Text Word] Or “Aortic Valve Stenosis” [Text Word) AND ((“Surgical Aortic Valve Replacement”/exp or SAVR [Text Word) OR (“Transcatheter Aortic Valve Replacement”/exp or TAVI [Text Word] OR TAVR|Text Word))

## Eligibility criteria

PRISMA guidelines and the ‘Population, Intervention, Comparison, Outcome, and Study Design (PICOS)’ scheme, were utilized to generate the eligibility criteria [14]. First, the literature considered eligible for inclusion comprised primary research that had undergone peer review, otherwise known as Randomized Controlled Trials (RCTs), and published after 2017 to those published till 2024. The target population included: (i) studies involving patients of aortic valve stenosis who underwent TAVI; (ii) patients with previously untreated symptomatic aortic valve disease, treated either with TAVI or SAVR; (iii) patients with adequate follow-up data available for assessment of clinical outcomes, (iv) studies reporting short-term and long-term complications, comparative efficacy, and overall survival rates, etc. The studies that were utilized investigated the impact on the efficacy and safety outcomes of the eligible patients. A summary of the inclusion and exclusion criteria for the review is provided in the table below. (Table 2).

**Table 2:** Description of eligibility criteria for systematic review.

Criteria	Inclusion	Exclusion
Study language	Literature published in the English language	All other languages
Study duration	All the studies published between 2017 and 2024	Studies older than 2017
Study design	Quantitative studies (randomized controlled trials)	Case series and case reports Protocols Reviews Observational studies (prospective and retrospective cohorts) Gray literature
Location	Global	–
Age	>18 years with stratification for high-, intermediate-, or low-risk surgical candidates as defined by clinical guidelines.	Pediatric patients
Target population	Patients with severe symptomatic aortic valve stenosis. Patients suitable for either transcatheter aortic valve implantation (TAVI) or surgical aortic valve replacement (SAVR). Patients with previously untreated symptomatic aortic valve disease, treated either with TAVI or SAVR	Studies focusing on patients with contraindications to either TAVI or SAVR, such as unsuitable anatomy or severe comorbidities making either procedure contraindicated. Studies on patients undergoing valve replacement for conditions other than aortic stenosis (e.g., infective endocarditis, congenital valve defects).
Follow-up	12 months and above	Less than 12 months
Context	Studies reporting at least one of the following clinical outcomes: mortality, stroke, rehospitalization, quality of life, or valve-related complications over short-term and long-term follow-up.	Studies with no quantitative data for pre- and post-treatment analysis. Non-comparative studies or those that do not provide separate outcome data for TAVI and SAVR groups.

## Selection process

A thorough examination of relevant articles and peer-reviewed journals was the foundation for developing the study methodology. The literature that satisfied our predetermined inclusion criteria was carefully examined, and the PICOS technique was utilized for a comprehensive analysis. The PICOS for the current study are summarized in the table below (Table 3):

**Table 3:** Description of PICOS (population, intervention, comparison, outcome, and study design) for the systematic review.

Aspect	Description
Population	Adults (≥18 years) with severe symptomatic aortic valve stenosis, eligible for either TAVI or SAVR.
Intervention	Transcatheter aortic valve replacement (TAVI).
Comparison	Surgical aortic valve replacement (SAVR).
Outcome	Short-term and long-term clinical outcomes, including mortality, stroke, rehospitalization, quality of life, valve-related complications (e.g., paravalvular leak, prosthetic valve dysfunction), etc.
Study design	Randomized controlled trials only.

After thoroughly selecting the literature, peer-reviewed journals with a strong impact factor were explored to decrease the risk of publication bias. All selected literature was uploaded to the screening software, Rayyan.ai, for primary and secondary screening of the literature [15]. Three researchers worked as collaborators to “include” or “exclude” eligible studies according to inclusion and exclusion criteria. A total of 7 studies were considered for final review and analysis. Studies that did not pass the eligibility for screening were put under “exclusion” or “dispute”. We created a team of 3 researchers for study selection to serve as tiebreakers for a disputed study. Exclusion reasons were put forward before excluding a study from the literature. Studies were excluded because 1: There was a problem with the population; 2: The study design was not ideal for our analysis; 3: The study measured the wrong outcomes; or 4: We found a high risk of bias. Sometimes, it was a combined effect of multiple reasons for exclusion.

## Data items

After finalizing the secondary screening process, we assessed the overall sample size (n=7)of the selected literature. To create a PRISMA flow chart that follows the rules of Preferred Reporting Items for Systematic Review and Meta-Analysis (PRISMA), we used articles from reputable journals and other sources (Figure 1) [16].

**Figure A:**
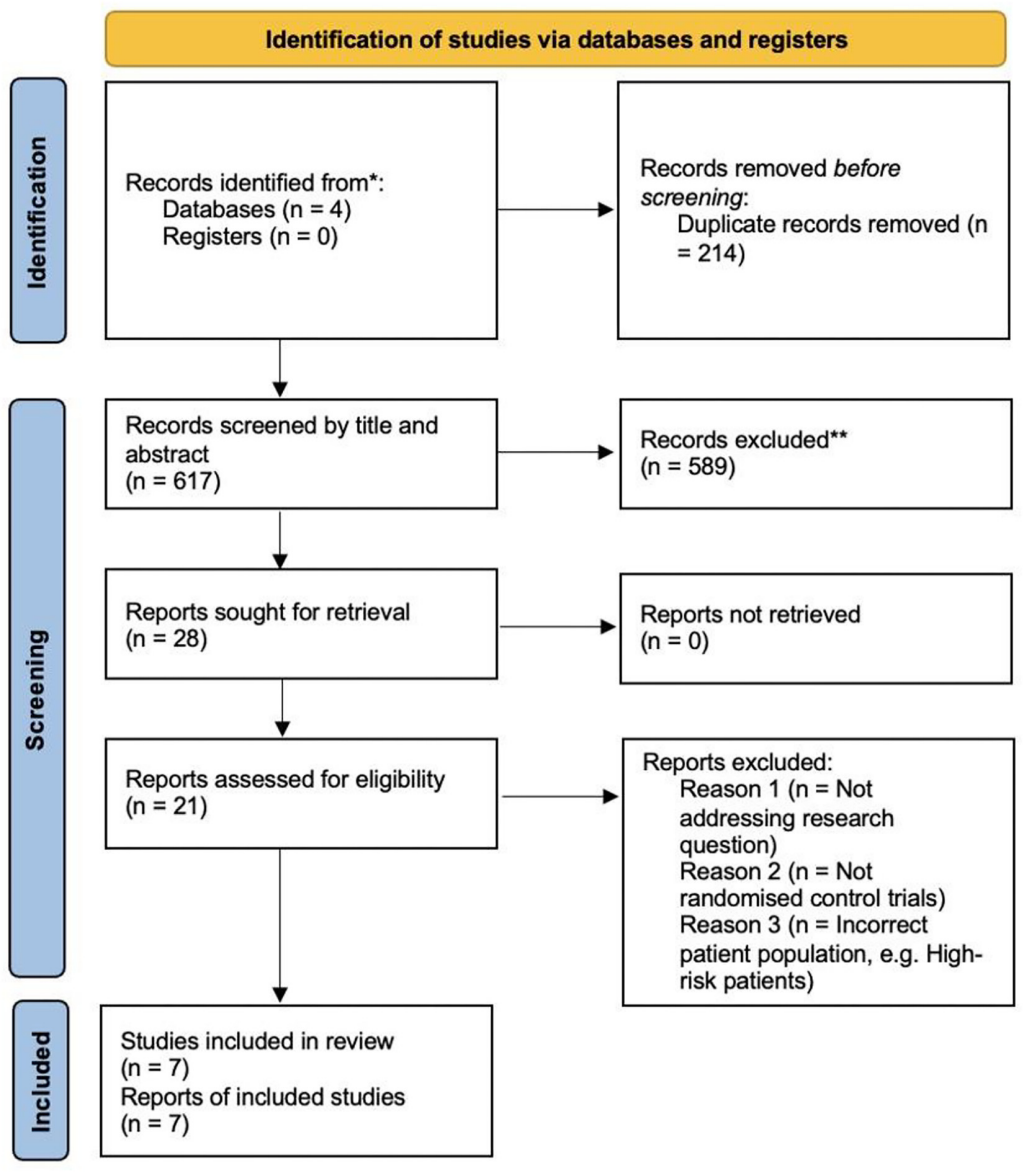
Preferred reporting items for systematic reviews and meta-analysis flow chart (n, number of studies).

Several measures were adopted to minimize bias in the analysis 1: a strict selection of the optimal research studies; 2: a requirement that peer reviewers declare any conflicts of interest; and 3: a preference for meta-analyses over traditional review articles. We purposefully excluded the narrative and systematic reviews to maintain the credibility of the study.

## Endpoints

The **primary** endpoints of this study include mortality and stroke (both disabling and non-disabling) at specified follow-up intervals (e.g., 30 days, 1 year, and long-term up to 5 years). When comparing the safety and effectiveness of TAVI with SAVR, these endpoints are significant. Mortality was defined as death from any cause, and stroke outcomes were further classified as debilitating or non-disabling, reflecting their impact on quality of life and clinical decision-making.

The **secondary** endpoints covered a broad variety of clinical and operative outcomes. Among these are cardiovascular death, myocardial infarction (MI), atrial fibrillation (Afib), transient ischemic attack (TIA), acute kidney injury (AKI), vascular complications, rehospitalization, major or life-threatening bleeding, prosthetic valve thrombosis or endocarditis, and permanent pacemaker implantation. Functional outcomes including an improvement in the New York Heart Association (NYHA) classification and procedural success rates like aortic valve reintervention were also examined.

## Assessment of research quality

*Systematic review:* We performed an in-depth review of bias in each primary study that was chosen for a quality assessment. For this, it was necessary to examine the demographics of the population, the features of the interventions, and the geographical area in which the study was carried out. Studies were selected based on predetermined qualifying criteria, which included study design, population characteristics, and results of interest. Following that, data extraction was carried out to acquire information about the total sample size and number of events in every study.

*Meta-analysis:* To evaluate the degree of bias in the research we selected, we used a range of digital and web-based resources. We conducted a thorough analysis of bias-prone domains [17]. To minimize bias, the following steps should be taken 1: create a random sequence; 2: keep allocations concealed; 3: blinding clinicians and participants; 4: blinding of outcome assessments; 5: address attrition bias; 6: avoid selective reporting; and 7: identify and mitigate other biases. Both continuous and dichotomous data were presented in all seven studies. The data were included in the statistical meta-analysis.

The included studies were pooled and statistically analyzed using the Comprehensive Meta-Analysis (CMA) software version 4. The program made it possible to compute effect sizes with associated 95 % CIs, such as mean difference (MD) for continuous outcomes and risk ratios (RR) for dichotomous data. Standardized mean differences (SMD) were determined for studies that reported heterogeneous data. Subgroup analyses and meta-regression were not performed because of the limited number of included RCTs and insufficient study-level data. Risk ratios were used to assess dichotomous outcomes (such as mortality and stroke), while mean differences were used to evaluate continuous outcomes (such as length of hospital stay).

All of the data in the investigation were available as continuous (Standard Mean Deviation (SMD), Confidence Interval (CI)), as well as dichotomous variables. Statistical heterogeneity was assessed using the I^2^ statistic, with values above 50 % indicative of substantial heterogeneity. The estimated within-study variance, or tau square (τ^2^), showed how variable impact sizes were among research. The number of independent comparisons needed to determine the pooled effect size was indicated by degrees of freedom (df). The chi-square (χ^2^) test determined if observed variations in effect sizes between studies were more than what would be predicted by pure chance. Heterogeneity was positive if the chi-square value was significant. The I-square (*I* ^2^) measures the percentage of overall variation that may be attributed to heterogeneity as opposed to random variation. High numbers indicated greater heterogeneity and provided the intuitive idea of the degree of discrepancy among study outcomes. The dataset used in the meta-analysis can be found in the results section.

## Risk of bias assessment

Risk of bias was evaluated using the Cochrane Risk-of-Bias 2 (RoB 2) tool. Two reviewers independently assessed the following domains 1: randomization process, 2: deviations from intended interventions, 3: missing outcome data, 4: outcome measurement, and 5: selection of reported results. Disagreements were resolved by consensus. Because blinding of participants is generally infeasible in TAVI vs. SAVR trials, this domain frequently resulted in “some concerns.” A complete risk-of-bias summary is provided in Table 4.

**Table 4:** Risk-of-bias assessments performed using the cochrane risk-of-bias 2 (RoB 2) tool for randomized controlled trials.

Study	D1: Randomization process	D2: Deviations from intended interventions	D3: Missing outcome data	D4: Outcome measurement	D5: Selection of reported results	Overall risk of bias
PARTNER 3	Low	Some concerns	Low	Low	Low	Some concerns
PARTNER 2	Low	Some concerns	Low	Low	Low	Some concerns
DEDICATE	Low	Some concerns	Low	Low	Low	Some concerns
NOTION	Low	Some concerns	Low	Low	Low	Some concerns
Evolut low risk	Low	Some concerns	Low	Low	Low	Some concerns
SURTAVI	Low	Some concerns	Low	Low	Low	Some concerns
UK TAVI	Low	Some concerns	Low	Low	Low	Some concerns

## Statistical analysis

The synthesis of results from several literature sources and research investigating the efficacy and safety outcomes in the sample of 7950 patients was made possible by this methodology. Using the relevant formulae, the log [RR] and its SE were determined from the retrieved data The natural logarithm of the risk ratio is represented by log (RR), which was calculated to derive pooled effect estimates and standard errors for dichotomous outcomes.Conversely, SE measures the precision or level of uncertainty in the log [RR] estimate. The *DerSimonian* and *Laird* random-effects model was then used to do the meta-analysis, which considers both within-study and between-study variability. With this method, an overall summary effect size in terms of the Relative Risk (RR)) and its associated confidence interval (CI=95 %) could be estimated.

Interpretation of statistical significance involved assessing whether the observed effect size was likely to have occurred by chance alone. The calculated effect size and its matching confidence interval (CI) were compared to achieve this. Interpretation of statistical significance was based on whether the 95 % confidence interval crossed 1.0 for risk ratios. Confidence intervals that included 1.0 were considered statistically non-significant.

## Ethical statement

As no new human subjects were recruited and no identifiable patient data were used, institutional review board (IRB) approval and informed consent were not required. All included trials reported obtaining ethical approval and participant consent in their original publications.

## Results

### Searching databases

A total of 831 studies were identified through database searches in PubMed, Embase, Medline, and Cochrane. After removing 214 duplicates, 617 studies remained for screening by title and abstract. Of these, 589 reports were excluded, leaving 28 studies sought for retrieval, which were all successfully retrieved. Following a detailed review assessing eligibility, 21 studies were excluded – 8 for not addressing the research question, 7 for not being RCTs, and 6 for focusing on high-risk patient populations. Ultimately, 7 studies met the inclusion criteria and were included in the final systematic review and meta-analysis after a PICO review.

### Study characteristics

Seven RCTs were included in the analysis, all of which were multicentered. Of these, five were multinational studies, while two were country specific (DEDICATE and UK TAVI). The total number of patients across the studies was 8,663, with individual study sample sizes ranging from 135 to 1,021 patients. The median number of patients per study was 692. The average age of participants varied from 73 to 81 years, with a median age of 79. On average, 58.3 % of the participants across all studies were male. Follow-up duration varied substantially across studies, ranging from 12 months (DEDICATE, UK TAVI) to 10 years (NOTION), which may contribute to observed heterogeneity in pooled mortality estimates.

### Statistical analysis

Meta-analysis was performed using a random effect model. Effect size was calculated for both primary and secondary outcomes in terms of risk ratios and log risk ratios. Heterogeneity in the analysis was assessed by calculating the I2 and measuring the prediction interval from the statistics. Moreover, I2 was also used as the measure of heterogeneity. We used the DerSimonian–Laird random-effects estimator, which remains appropriate for meta-analyses with a small number of studies and is widely used in prior TAVI–SAVR evidence syntheses. Alternative estimators such as REML were considered; however, given only seven RCTs and inconsistent reporting of study-level moderators, more complex variance estimators or prediction intervals would yield unstable and clinically uninformative estimates.

### Risk of bias

Overall, the seven included RCTs demonstrated low risk of bias across most evaluated domains. The primary methodological limitation was lack of blinding, which is inherent to procedural comparisons between TAVI and SAVR. Missing data, outcome measurement, and selective reporting domains were consistently judged as low risk. Formal assessment of publication bias using funnel plots or asymmetry testing was not performed because fewer than 10 studies were included, limiting the interpretability and statistical reliability of these methods.

### Primary outcome

Our meta-analysis found that hospitalized patients with symptomatic aortic valve stenosis who were treated with TAVI had a reduced likelihood of mortality compared to those who underwent SAVR (relative risk [RR]: 0.88, 95 % CI: 0.62–1.25, p=0.49). Although the data suggests a lower mortality rate with TAVI, these findings were not statistically significant. Additionally, there was considerable heterogeneity in the results (I^2^: 80 %), indicating variability in how different studies assessed mortality outcomes between TAVI and SAVR.

As shown in Figure B, 5 of the 7 trials (Dedicate Trial, NOTION, Evolut Low Risk, SURTAVI, and UK Tavi) provided results favoring TAVI in terms of reduced likelihood of mortality. However, only 2 of those trials favoring TAVI (Dedicate Trial and Evolut Low Risk) provided statistically significant results in terms of mortality outcomes. Mortality outcomes in the dedicate trial and Evolut Low Risk trial were RR: 0.52, 95 % CI: 0.34–0.78, p=0.003, and RR: 0.68, 95 % CI: 0.46–0.99, p=0.04, respectively. PARTNER 3 and PARTNER 2 trials favored SAVR in terms of reduced likelihood of mortality (RR: 1.41, 95 % CI: 0.84–2.10, p=0.23, and RR: 1.41, 95 % CI: 1.18–1.69, p<0.001, respectively).

**Figure B:**
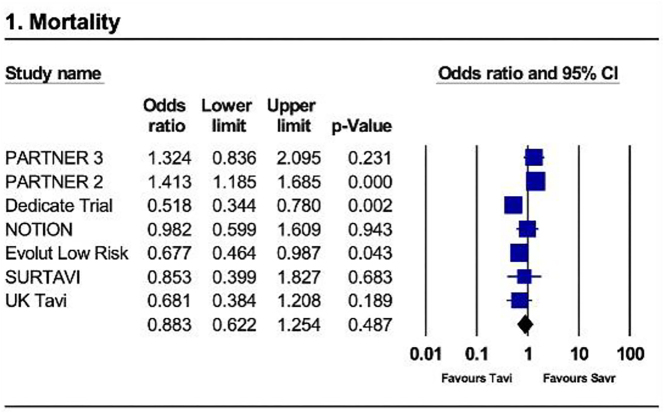
Forest plot for mortality outcomes.

### Secondary outcomes

TAVI compared to SAVR was associated with an increased risk for post-procedure MI and PPM insertion (RR: 1.11, 95 % CI: 0.80–1.53, p=0.53, i2: 25 %, and RR: 2.19, 95 % CI: 1.54–3.11, p<0.001, i2: 80 %, respectively). PPM insertion showed high heterogeneity (80 %), with substantial variability between studies. In contrast, the incidence of MI showed low heterogeneity (0 %), with most studies showing similar risk ratios and a pooled result indicating no significant difference between TAVI and SAVR (Figure C).

**Figure C:**
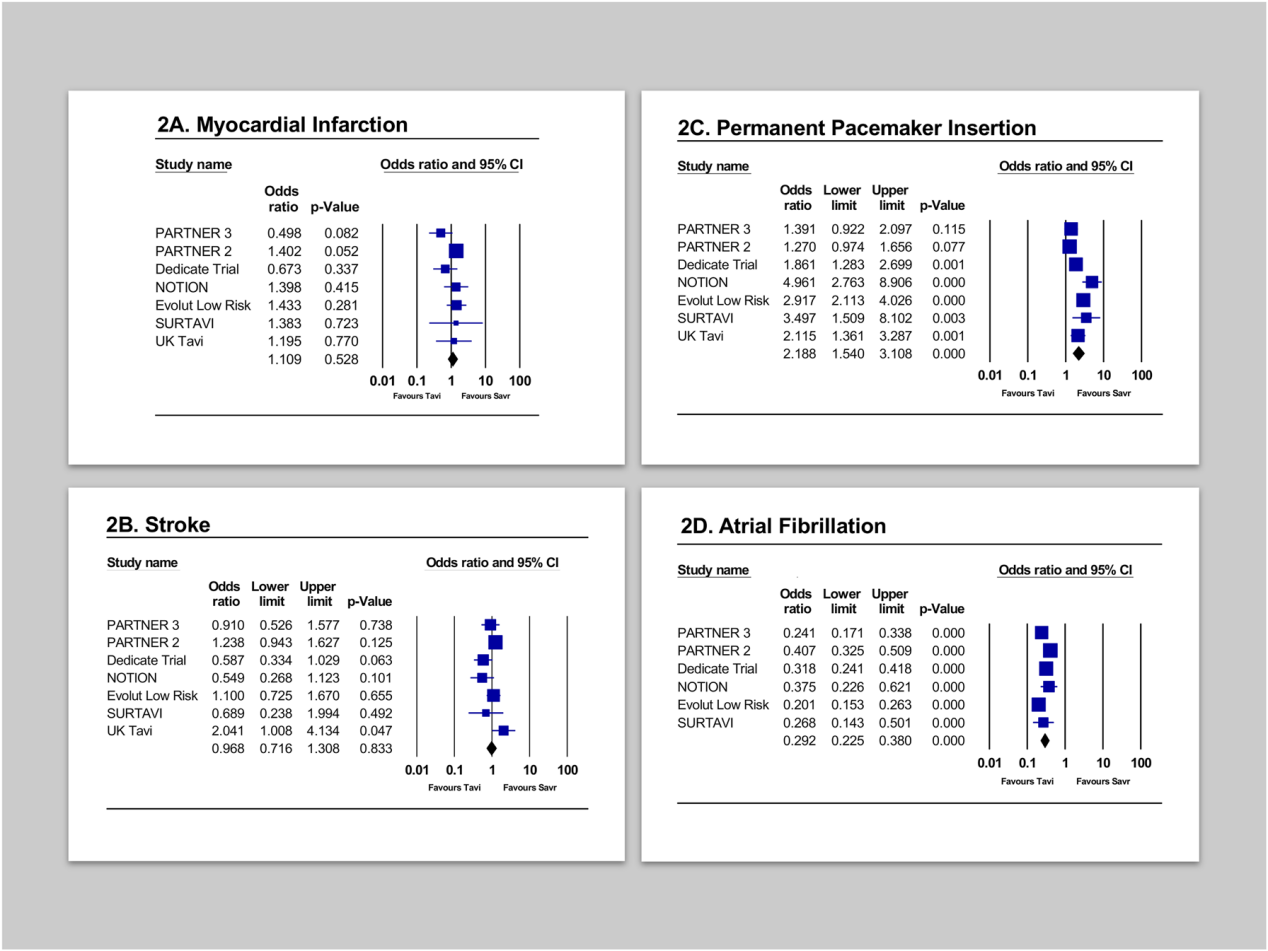
Forest plots for myocardial infarction (2A), permanent pacemaker insertion (2B), stroke (2C), and atrial fibrillation (2D).

The risk for stroke complications (Figure C) was increased with TAVI compared to SAVR (relative risk RR: 0.97, 95 % CI: 0.72–1.31, p=0.83). The risk for stroke was greater with SAVR compared to TAVI (RR: 2.04, 95 % CI : 0.72–1.31, p=0.83). Stroke complications also had considerable heterogeneity (I2: 54 %).

TAVI was associated with a significantly lower risk of atrial fibrillation compared to SAVR (RR: 0.29, 95 % CI 0.23–0.38, p<0.001; I^2^=73 %). The risk for rehospitalization was greater with SAVR compared to TAVI (RR: 1.01, 95 % interval CI: 0.66–1.55, p=0.95). Results for rehospitalization (Figure D) were statistically insignificant and also had considerable heterogeneity (I2: 80 %).

**Figure D:**
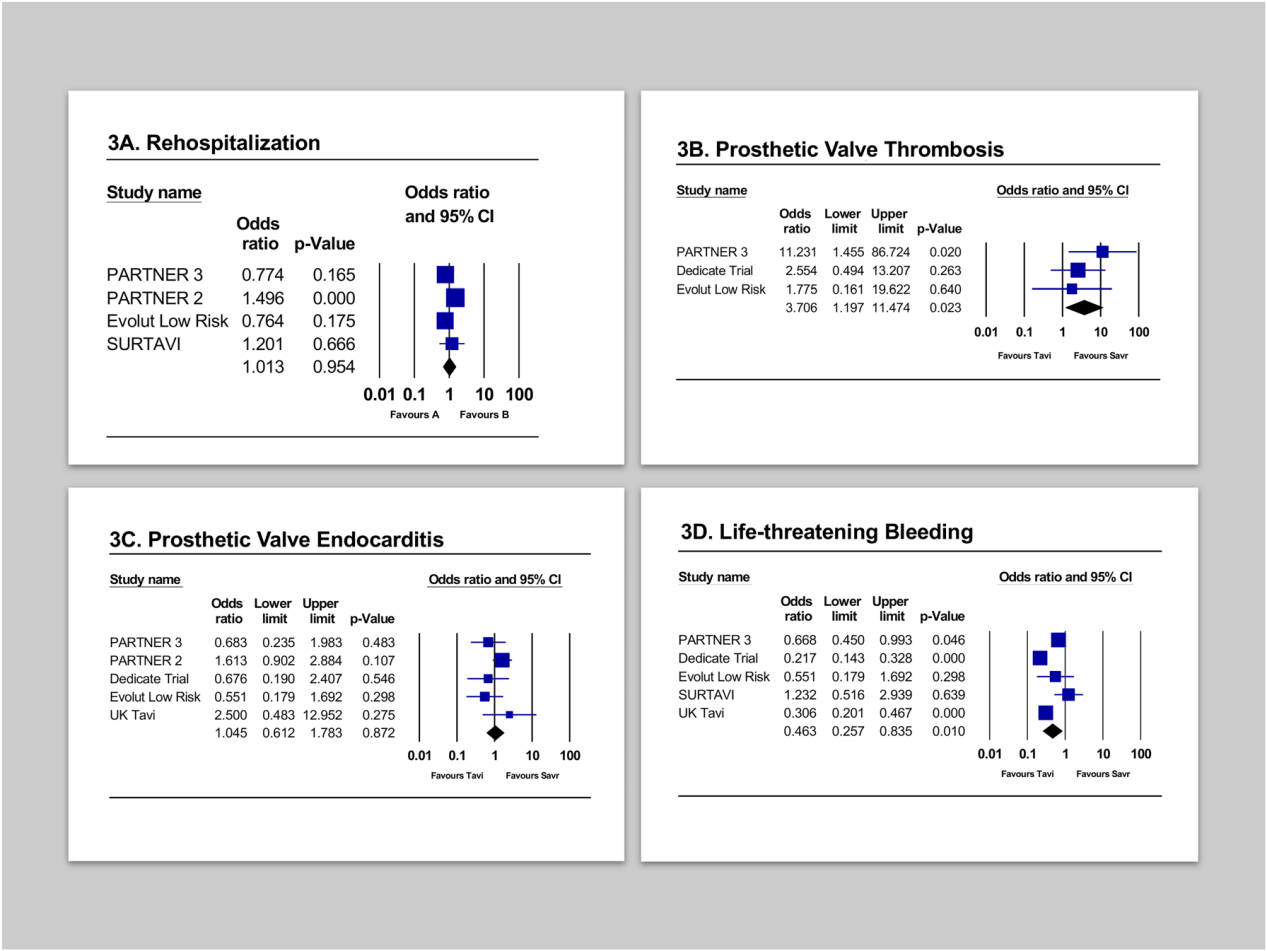
Forest plots for rehospitalization (3A), prosthetic valve thrombosis (3B), prosthetic valve endocarditis (3C), and life-threatening bleeding (3D).

TAVI was associated with a significantly higher risk of prosthetic valve thrombosis (Figure D) compared to SAVR (RR: 3.71, 95 % CI: 1.20–11.47, p: 0.02; I^2^: 0 %). There was no significant difference between TAVI and SAVR in the incidence of prosthetic valve endocarditis (RR: 1.05, 95 % CI: 0.61–1.78, p: 0.87; I^2^: 26 %). TAVI was associated with a reduced risk of life-threatening bleeding compared to SAVR (RR: 0.46, 95 % CI: 0.26–0.84, p: 0.01; I^2^: 83 %) (Table 5).

**Table 5:** Baseline characteristics of randomized controlled trials included in meta-analysis.

Variable	Intervention	PARTNER 3	PARTNER 2	Dedicate trial	NOTION	Evolut low risk	SURTAVI	UK tavi
Publication (Year)		2019	2020	2024	2024	2017	2022	2022
Location		Multicenter (USA)	USA and Canada	Multicenter (Germany)	Denmark and Sweden	Multinational	Multicenter (87 centers)	UK
No. of patients	TAVI	496	1,011	701	145	730	864	458
	SAVR	454	1,021	713	135	684	796	455
Follow-up (months)	TAVI	NA	NA	12	120	48.4	24	12
	SAVR	NA	NA	12	120	48.1	24	12
Design	TAVI	Multinational, randomized,noninferiority clinical trial	Multinational, randomized, clinical trial	Randomized non-inferiority trial	RCT	RCT	Multinational, randomized, noninferiority clinical trial	RCT
	SAVR	Multinational, randomized,noninferiority clinical trial	Multinational, randomized, clinical trial		RCT	RCT	Multinational, randomized, noninferiority clinical trial	RCT
Age, years	TAVI	73.3 ± 5.8	81.5 ± 6.7	74.3 ± 4.6	79.2 ± 4.9	74	79.9 ± 6.2	81 (79–84)
	SAVR	73.6 ± 6.1	81.7 ± 6.7	74.6 ± 4.2	79.0 ± 4.7	74	79.7 ± 6.1	81 (78–84)
Men, %	TAVI	67.5 %	54.2 %	56 %	53.8 %	63.6 %	57.6 %	53.9 %
	SAVR	71.1 %	54.8 %	57.3 %	52.6 %	65.9 %	55.0 %	53.2 %
Non-white/ethnic group, %	TAVI	7.7 %		N/A		Not specified	2.4 %	
	SAVR	9.9 %		N/A		Not specified	4.6 %	
BMI (kg/m^2^)	TAVI	30.7 ± 5.5	28.6 ± 6.2	28.1 (25.3–31.9)	N/A		27.8 ± 5.0	27.1 (24.0–30.5)
	SAVR	30.3 ± 5.1	28.3 ± 6.2	28.1 (25.4–31.2)	N/A		28.0 ± 4.8	27.7 (24.7–31.2)
STS mean score, %	TAVI	1.9 ± 0.7	5.8 ± 2.1	1.8 (1.2–2.4)	3.0 ± 1.7	2.0 ± 0.7	4.4 ± 1.5	2.6 (2.0–3.5)
	SAVR	1.9 ± 0.6	5.8 ± 1.9	1.9 (1.2–2.5)	3.0 ± 1.7	1.9 ± 0.7	4.5 ± 1.6	2.7 (2.0–3.4)
NYHA class III/IV, %	TAVI	31.2 %	77.3 %	46.2	83.7 %	III: 24.8 %, IV: 0.1 %	54.6 % (III), 5.6 % (IV)	40.3 %
	SAVR	23.8 %	76.1 %	45.6	80.6 %	III: 27.8 %, IV: 0.4 %	51.6 % (III), 6.5 % (IV)	45.2 %

## Discussion

Across low- and intermediate-risk patients, TAVI and SAVR demonstrated comparable all-cause mortality, with no statistically significant difference in pooled outcomes. Secondary outcomes showed a divergent profile, with TAVI associated with higher rates of PPM implantation and conduction disturbances but lower rates of atrial fibrillation and major bleeding. AS increases the risk of mortality, and patients who exhibit symptoms often require valve replacement [18]. SAVR has long been the preferred treatment for patients suffering from symptomatic severe AS. Nonetheless, TAVI has surfaced as a viable alternative, particularly for those with an increased surgical risk [19]. Given that it is a newer treatment approach, there is a limited amount of data on the long-term outcomes associated with TAVI. Various RCTs and observational studies have indicated that TAVI has certain benefits over SAVR, especially regarding survival rates in high surgical risk patients [20]. Consequently, many international guidelines now endorse TAVI for high risk AS, recent 2025 ESC valvular heart disease guidelines continue to recommend TAVI as the preferred approach for high-risk patients and as an appropriate option for selected intermediate- and low-risk individuals following Heart Team evaluation. This aligns with the evolving evidence base reflected in the trials included in our review. [21]. However, there is still debate surrounding the optimal treatment for patients with lower surgical risk. In this meta-analysis, we considered RCTs that involved patients categorized as low or intermediate surgical risk, based on the commonly utilized STS-PROM risk assessment score. The SURTAVI and PARTNER-2 trials focused on intermediate risk patients, with average STS-PROM scores of 4.5 and 5.8, respectively. All other trials exclusively included low-risk patients, exhibiting STS-PROM scores below 4. Additionally, we included the recently released DEDICATE trial and the 10-year follow-up findings from the NOTION trial in our meta-analysis, ensuring that the most recent data was evaluated.

Our meta-analysis indicated that patients with symptomatic AS who received TAVI exhibited a reduced mortality rate compared to those who underwent SAVR. However, this observation did not reach statistical significance (p=0.49). Only two trials, the DEDICATE and EVOLUT LOW-RISK trials, showed a statistically significant mortality benefit for TAVI over SAVR. These trials had mean STS-PROM scores of 1.8 and 2.0, respectively. It is important to highlight that the DEDICATE trial involved patients who had valve replacement surgeries from 2017 to 2022, a significant portion of which occurred during the pandemic. The results indicated a notably higher mortality rate among low-risk surgical patients undergoing SAVR, which may be attributable to the impact of the COVID pandemic, which has been shown to considerably elevate mortality risk, particularly in cardiac surgeries [22]. Examining the EVOLUT LOW-RISK trial, all patients receiving TAVI were implanted with a self-expanding, supra-annular valve (Core Valve/Evolut platform), featuring tall commissures specifically designed to enhance hemodynamics and reduce bioprosthetic leaflet stress. This design has been demonstrated to reduce structural valve deterioration, which correlates with lower mortality rates [23].

Our results are consistent with earlier studies, which showed that in in moderate and high-risk patients, there was no significant difference between TAVI and SAVR, in terms of mortality. For example, a meta-analysis including data of 2818 patients, reported similar mortality rates between TAVI and SAVR [24]. Another study showed that in patients with a low surgical risk, TAVI had better mortality outcomes at 1 year. However, on long term follow-up, there was no difference in the primary outcome [25]. Another meta-analysis also reported a mortality benefit for TAVI vs. SAVR in the short term. Interestingly, this benefit was reversed at 24 months, favoring SAVR [26]. This highlights the need for further studies evaluating the longer-term outcomes of TAVI vs. SAVR, particularly as more and more young patients with severe AS are undergoing TAVI.

In our meta-analysis, secondary outcomes from the trials were also analyzed, which yielded important findings. TAVI was linked to a higher incidence of permanent pacemaker implantation during the follow-up period. Additionally, TAVI was associated with an increased risk of aortic valve re-intervention, new-onset bundle branch block, and vascular site complications. Patients undergoing TAVI had a higher rate of myocardial infarction, although this association did not reach statistical significance. There was no statistically significant relationship between TAVI and stroke incidence in the overall meta-analysis. However, the PARTNER 3 and DEDICATE trials reported a reduced incidence of stroke in the TAVI cohort. A significant reduction in atrial fibrillation incidence is also observed in the TAVI group. Together, these findings indicate that TAVI and SAVR have distinct side effect profiles. This may be attributed to the difference in approach to valve replacement [27], 28].

Other studies have reported similar outcomes. For example, a study has demonstrated no difference in the incidence of stroke in TAVI and SAVR [29]. However, they did report an increased incidence of permanent pacemaker insertion and vascular complications in TAVI patients. In contrast, complications such as new-onset atrial fibrillation and acute kidney injury occurred less often in patients who underwent TAVI as compared to SAVR [29].

Improved valve delivery systems, enhanced procedural expertise, and a decreased risk profile of TAVI patients have led to a reduction in vascular and bleeding complications over time. Newer generation THVs (transcatheter heart valves) have contributed to a decreased incidence of vascular site complications and PVL in TAVI patients [30]. A large registry-based study from the US, including data of 34,893 patients who underwent TAVI via transfemoral access, reported that 9.3 % of the patients experienced vascular complications while 7.6 % had an in-hospital bleeding event. Interestingly, they also reported that from 2011 to 2016, the rates of both vascular complications and bleeding events decreased [31].

In comparison, the rates of post-TAVI PPM insertion and conduction abnormalities remain high, even with newer THVs. High degree AV block has been the main indication for post-TAVI PPM insertion, followed by other bradyarrhythmia [32]. The increased incidence of conduction abnormalities in TAVI patients can be explained by the proximity of the aortic valve to the conduction system [33]. New-onset bundle branch block and PPM insertion are associated with an elevated all-cause mortality and hospitalization in TAVI patients [34]. A large retrospective cohort study, including 9,785 patients, reported that post-TAVI PPM insertion was associated with a higher composite of mortality and heart failure admission at 1 year follow-up [35]. The type of bioprosthetic valve used may also have an effect on the rate of PPM insertion. Implantation with the self-expandable CoreValve has been associated with a significantly elevated incidence of PPM insertion than the balloon-expandable Sapien Valve [36]. Male sex, previous history of AV conduction abnormalities, intra-procedural AV block have also been identified as predictors of post-TAVI PPM insertion [37].

As TAVI is now preferred even in younger patients of AS with a low surgical risk, long-term valve durability is an important issue to consider. Data regarding long-term durability of surgical bioprosthesis is already established [38]. However, long-term data for TAVI is an area of ongoing research. The recently published 10-year data from the NOTION trial is promising [7]. It shows that the risk of moderate to severe structural valve deterioration (SVD) and bioprosthetic valve failure (BVF) is not significantly different between (TAVI and SAVR). The patients randomized to the TAVI group received self-expandable CoreValve bioprosthesis. The PARTNER-2 trial, with a follow-up duration of 5 years, reported no difference in mortality. However, aortic valve re-intervention was more common after TAVI than SAVR [11]. The recently published 5 year follow-up data from the PARTNER-3 trial showed similar rates of BVF in both groups [10]. All of these studies with longer follow-up duration were included in our meta-analysis.

A study which analyzed outcomes in patients with tricuspid and bicuspid aortic valves (BAV) undergoing TAVI, concluded that there was no significant difference in the rates of all-cause mortality and stroke at 30 days and 1 year [39]. Results from other studies are also promising, showcasing a high procedural success rate with TAVI as well as a low incidence of major complications [40]. However, there is a paucity of data comparing the outcomes of TAVI and SAVR in patients with BAV. This is because most of the landmark trials comparing TAVI and SAVR outcomes had excluded patients with BAV. Thus, further studies that analyze the long-term outcomes of BAV-TAVI are required.

Our meta-analysis included 7 different RCTs. There were variations across these studies in terms of population characteristics, primary and secondary outcomes evaluated and follow-up duration. Follow-up period ranged from 1 year in the UK TAVI and DEDICATE trials to a maximum follow-up of 10 years in the NOTION trial. Differences in follow-up duration across trials represent a key source of heterogeneity and should be considered when interpreting pooled mortality results, as longer-term studies such as NOTION may capture structural valve deterioration that short-term studies cannot. Data of some secondary outcomes like new-onset bundle branch block, atrial fibrillation, vascular site complications and endocarditis was not present for all the trials. There was a lack of data on patient reported outcomes like functional status, quality of life and symptom relief. Furthermore, we did not include a detailed sub-group analysis in our study. Applicability of these findings to all healthcare settings should be interpreted with caution. Differences in study design, valve technology, patient selection, and industry involvement may contribute to the observed heterogeneity and reflect residual confounding even within large RCTs. These factors likely influence external validity and underscore the need for ongoing independent long-term data.

Despite these limitations, our study contributes to a growing body of research on outcomes of TAVI vs. SAVR in patients with lower surgical risk. In conclusion, there was no significant difference in all-cause mortality between TAVI and SAVR in intermediate and low risk patients. Secondary outcomes were also analyzed, which showed that TAVI was associated with an increased risk of PPM insertion, vascular site complications, new-onset bundle branch block and aortic valve re-intervention. TAVI was associated with a decreased risk of atrial fibrillation as compared to SAVR. There was no statistically significant association of TAVI with stroke (disabling and non-disabling), MI, post-TAVI endocarditis. Technological enhancement and increased procedure expertise may improve outcomes further in TAVI patients. As TAVI is becoming increasingly favored, even in low-risk patients, future studies focusing on long-term effects of TAVI are required.
